# Intra-voxel incoherent motion biomarker repeatability in healthy volunteers and sensitivity to chemoradiotherapy-induced changes in patients with uterine cervical cancer

**DOI:** 10.3389/fonc.2025.1633456

**Published:** 2025-11-26

**Authors:** Damien J. McHugh, Anubhav Datta, Michael J. Dubec, David L. Buckley, Ross A. Little, Michael Berks, Susan Cheung, Kate Haslett, Lisa Barraclough, Catharine M. L. West, Ananya Choudhury, Peter Hoskin, James P. B. O’Connor

**Affiliations:** 1 Christie Medical Physics and Engineering, The Christie National Health Service (NHS) Foundation Trust, Manchester, United Kingdom; 2 Division of Cancer Sciences, The University of Manchester, Manchester, United Kingdom; 3 Clinical Radiology, The Christie National Health Service (NHS) Foundation Trust, Manchester, United Kingdom; 4 Biomedical Imaging, University of Leeds, Leeds, United Kingdom; 5 Clinical Oncology, The Christie National Health Service (NHS) Foundation Trust, Manchester, United Kingdom; 6 Division of Radiotherapy and Imaging, Institute of Cancer Research, London, United Kingdom

**Keywords:** biomarkers, diffusion magnetic resonance imaging, image processing, intra-voxel incoherent motion, model comparison, uterine cervical neoplasms, radiotherapy

## Abstract

Intra-voxel incoherent motion (IVIM) biomarkers require validation for translation into clinical practice. This work evaluates repeatability and sensitivity to treatment of IVIM biomarkers in the uterine cervix, and assesses suitability of the IVIM model. Six healthy volunteers underwent two scans to evaluate repeatability. Eight patients with stage IIB-IVA cervical squamous cell carcinoma were scanned pre-treatment, and at weeks 3 and 5 into treatment. IVIM and apparent diffusion coefficient (ADC) model fits were compared using the corrected Akaike information criterion (AIC_c_). Tissue diffusion coefficient, *D*, perfusion signal fraction, *f*, and *p*_IVIM_, the fraction of voxels better described by the IVIM model, were measured. ADCs calculated with minimum *b*-values of 0 (ADC*_b_*_0_) and 150 s/mm^2^ (ADC*_b_*_150_) were compared with *f* to assess sensitivity to perfusion. Model preference maps qualitatively reflected physiological characteristics of different tissues. Healthy cervix within-subject coefficients of variation were 8% (*D*), 15% (*f*), and 12% (*p*_IVIM_). Tumour *D* increased from baseline to week 3 (*p* = 0.02). Baseline *p*_IVIM_ showed large inter-patient variability (range: 0.13-0.68), which persisted throughout treatment. The difference between ADC*_b_*_0_ and ADC*_b_*_150_ correlated with *f* (repeated measures correlation coefficient r=0.76, *p* = 0.002). IVIM biomarkers are repeatable in healthy cervix tissue. Tumour *D* is sensitive to early therapy-induced changes. The IVIM model is not favoured in all tumour voxels, indicating the presence of heterogeneous tumour microenvironments. ADC calculated using *b* = 0 s/mm^2^ can be influenced by a perfusion-dependent bias. Not all tumour voxels are best described by the IVIM model. ADC in cervical tumours can suffer from perfusion-dependent bias.

## 1 Introduction

Uterine cervical cancer poses a significant global health challenge, particularly in developing regions where access to preventive measures and screening are often limited. Locally advanced cases frequently necessitate concurrent chemoradiation, aiming to achieve optimal local control and minimize the risk of recurrence (1).

There is a need for validated imaging biomarkers (2) to assess the early response of cervical tumours to therapy. Diffusion-weighted (DW) MRI is a functional imaging technique which provides various quantitative biomarkers that have potential to evaluate tumour response to therapy (3). Modelling the DW signal as a mono-exponential decay with *b*-value yields the apparent diffusion coefficient (ADC), the simplest quantitative biomarker to measure from DW-MRI, with several studies reporting values in cervical tumours (3–7).

Intra-voxel incoherent motion (IVIM) is a DW-MRI method that uses a bi-exponential decay model to separate the effects of tissue diffusion and capillary blood flow; as such, it provides more specific information about tumour microstructure and microvasculature than ADC, yielding parameters such as the tissue diffusion coefficient, *D*, and the perfusion signal fraction, *f* (8). IVIM does not require gadolinium-based contrast agents, and parameters have distinguished cervical tumours from non-malignant uterine tissues (9), and distinguished between cervical tumour histological subtypes and/or grades (10–12). Several studies have investigated the ability of IVIM biomarkers to predict and assess cervical tumour treatment response (13–17). Recent studies have also shown the utility of IVIM in predicting parametrial invasion (18), and shown that combined IVIM and FDG PET can identify lymphovascular invasion (19) and treatment resistance (20) in cervical cancer.

IVIM biomarkers require validation if they are to be translated into clinical practice (2). In particular, the IVIM model may not be applicable in all tumour regions (12) and model suitability may vary over the course of therapy. Previous work has shown spatial and temporal variation in model suitability for non-IVIM DW-MRI models (21), but this type of analysis has not yet been performed in IVIM studies of cervical cancer.

As IVIM requires longer scan times and more complex model fitting than ADC, IVIM biomarkers must provide additional utility over ADC, and potential bias when using the simpler ADC biomarker must be understood. In particular, several studies reporting ADC in cervical tumours use *b* = 0 s/mm^2^ as the lowest *b*-value (3–7), which is expected to make such ADCs sensitive to perfusion effects (22). As well as increasing ADC values, this may impact the evaluation of treatment-induced ADC changes if treatment affects both tumour tissue and vasculature.

Here we employ a model comparison framework to assess the spatial and temporal variability in suitability of the IVIM model, investigating both healthy uterine tissue and uterine cervical tumours (23). IVIM repeatability is evaluated through test-retest scanning of healthy volunteers. The sensitivity of IVIM to therapy-induced changes, and the impact of perfusion on ADC measurements, is assessed in patients with cervical cancer.

## 2 Methods

Research ethics committee approval was obtained. Fully informed written consent was obtained from all participants. Power calculations were not performed for this feasibility study.

### 2.1 Study design

Healthy volunteers were recruited between February 2021 and July 2022, and were scanned in two separate imaging sessions to evaluate repeatability. Patients with locally advanced cervical cancer (stages IIB-IVA) were recruited between April 2021 and July 2022. All patients underwent standard of care treatment: weekly cisplatin chemotherapy prescribed at 40 mg/m^2^, and combined chemoradiation/brachytherapy prescribed to reach a final dose of 85–90 Gy equivalent dose in 2 Gy fractions (EQD2) to the macroscopic tumour. Patients were scanned at up to three time points: pre-treatment, week 3, and week 5 of treatment. Imaging at week 3 (mid-chemoradiotherapy) and week 5 (end of chemoradiotherapy), allowed assessment of treatment-induced changes before the start of brachytherapy.

### 2.2 Data acquisition

Imaging was performed on a 1.5 T Philips Ingenia MR-RT system (Philips Healthcare). For patients, intra-vascular administration of 20 mg of Buscopan was performed subject topatient preference. Four patients received the drug. All participants were encouraged to follow a urinary bladder double-void protocol, aimed at minimising urinary bladder motion.

The same multiparametric MR protocol was used for all patient scans, including: a sagittal T2-weighted anatomical sequence; a sagittal pulsed-gradient spin-echo (PGSE) echo-planar imaging diffusion sequence with b-values = 0, 20, 40, 60, 80, 100, 150, 300, 500, 800 s/mm^2^, 4 signal averages, TR = 2800 ms, TE = 61 ms, voxel size = 2.9 x 2.9 x 6.0 mm^3^, slices = 20, SENSE = 2, fat suppression = SPIR, scan time = 05:16. Identical T2-weighted and DW-MRI sequences were used for all healthy volunteer scans.

### 2.3 Model fitting, model comparison and biomarker derivation

IVIM and ADC models were fitted voxel-wise to data at all *b*-values. Model fits were compared using the corrected Akaike information criterion (AIC_c_) (24); the fit with the lower AIC_c_ provides a statistically better characterisation of the signal, and is taken as the ‘preferred’ or ‘favoured’ model for that voxel (21) (**Supplementary Figure S1**). AIC balances model complexity against goodness-of-fit, and has been used in several studies comparing signal models for different MR techniques (25–28); the AIC_c_ is appropriate when the number of data points is small relative to the number of estimated model parameters, as is the case here. Throughout, references to one model being preferred/favoured is used as shorthand for that model providing a better characterisation of the signal decay based on having a lower AIC_c_. ADC-favoured voxels are expected to reflect regions with a single diffusion component, while IVIM-favoured voxels are expected to reflect regions with a significant perfusion component. The ADC obtained from this fitting is termed ADC*_b_*_0_.

The IVIM model was fitted using a segmented approach (29) with a range of *b*-value cut-off values, selecting the final parameters from the fit with the lowest sum of squared residuals. IVIM parameter maps (tissue diffusion coefficient, *D*, and perfusion signal fraction, *f*) were generated, along with model preference maps showing which model was favoured in each voxel. ADC maps were also generated by fitting a mono-exponential decay to *b* = 150, 300, 500, 800 s/mm^2^ data points; lower *b*-values were excluded from this fit, termed ADC*_b_*_150_, to reduce perfusion effects (30). All DICOM data were converted to Analyze format (31) and fitting was performed using the lmfit package (version 1.2.2) in Python (32).

Regions of interest (ROIs) in the uterine cervix and uterine body (contouring the myometrium and avoiding the endometrial lining) were defined for healthy volunteers. Whole-tumour and uterine body ROIs were defined for patients. Tumours were delineated at their outer margins, excluding any macroscopic necrotic/cystic areas. During treatment, likely post-radiation fibrotic regions were avoided, with ROIs limited to viable tumour. All ROIs were defined by one radiologist (AD, 7 years’ experience in female pelvic imaging) on *b* = 800 s/mm^2^ images, while referring to T2-weighted images. Median *D* and *f* were obtained over all ROI voxels, along with the fraction of voxels in which the IVIM model was favoured, termed *p*_IVIM_.

### 2.4 Healthy volunteer repeatability and patient comparison

Repeatability was quantified from the healthy volunteer uterine cervix and uterine body data using the within-subject coefficient of variation (wCV) (33). Differences in parameters between healthy volunteers and patients were evaluated using unpaired t-tests after testing for normality using Q-Q plots and the Shapiro-Wilk test (Pingouin Python package, version 0.5.4). Healthy volunteer uterine cervix parameters were compared with those from patient tumours; uterine body parameters for both groups were compared. For these comparisons, either single median values or means of repeat median values were used for healthy volunteers, and pre-treatment median values were used for patients.

### 2.5 Sensitivity to treatment-induced biomarker changes in patient tumours

Patient longitudinal data were used to assess the sensitivity of IVIM parameters and ADC_b150_ to detect early therapy-induced changes. Normality was assessed using Q-Q plots and the Shapiro-Wilk test, and sphericity (i.e. equal variance of differences) was assessed using Mauchly’s test. Longitudinal changes were analysed using repeated measures ANOVA, followed by two *post-hoc* pairwise t-tests with Bonferroni correction: baseline vs. week 3, and baseline vs. week 5 (Pingouin Python package, version 0.5.4). Bonferroni correction was applied by multiplying *p*-values by the number of comparisons (in this case 2). Throughout, *p* < 0.05 was taken to indicate statistical significance.

### 2.6 Influence of model preference on tumour IVIM and ADC parameters

To evaluate if median IVIM parameters were affected by the inclusion of voxels where the ADC model is favoured over IVIM, whole-tumour median *D* and *f* were compared with median *D* and *f* calculated from the subset of voxels where IVIM is favoured. For each approach to obtaining summary statistics, median values were compared separately for baseline, week 3, and week 5, using a paired t-test or Wilcoxon signed-rank test, depending on normality assessment using Q-Q plots and the Shapiro-Wilk test. In addition, median ADC_b0_ and ADC_b150_ were compared to *D* for voxels where the ADC model was preferred, to investigate the impact of analysis model on diffusivities in the same voxels. Median values were compared separately for baseline, week 3, and week 5, using a paired t-test or Wilcoxon signed-rank test, depending on normality assessment using Q-Q plots and the Shapiro-Wilk test.

### 2.7 Influence of perfusion on tumour diffusivities

To assess the influence of perfusion on ADC, tumour ADC_b150_ was compared with ADC_b0_ for all patients and time points: the median of voxel-wise differences, δADC = ADC_b0_ - ADC_b150_, was correlated with *f*, testing the hypothesis that the inclusion of low *b*-values in ADC calculations has a greater influence on ADC for tumours in which the perfusion fraction is higher. In addition, ADC_b0_, ADC_b150_, and *D* were directly correlated with *f*, to investigate the influence of *f* on different methods of calculating diffusivities. To account for patients having multiple measurements, repeated measures correlations were used (Pingouin Python package, version 0.5.5).

## 3 Results

Six healthy volunteers (mean ± standard deviation [s.d.] age: 26 ± 1 years; all pre-menopausal) were scanned in two sessions a median of 8 days apart (range: 7–70 days). Eight patients (age: 47 ± 19 years; four pre-menopausal; **Supplementary Table S1**) were scanned, with pre-treatment scans a median of 6 days before starting treatment (range: 3–27 days). Six patients were scanned at all three time points, and two missed the week 3 scan due to scheduling difficulties during COVID-19 restrictions.

### 3.1 Model comparison

**Figure 1:**
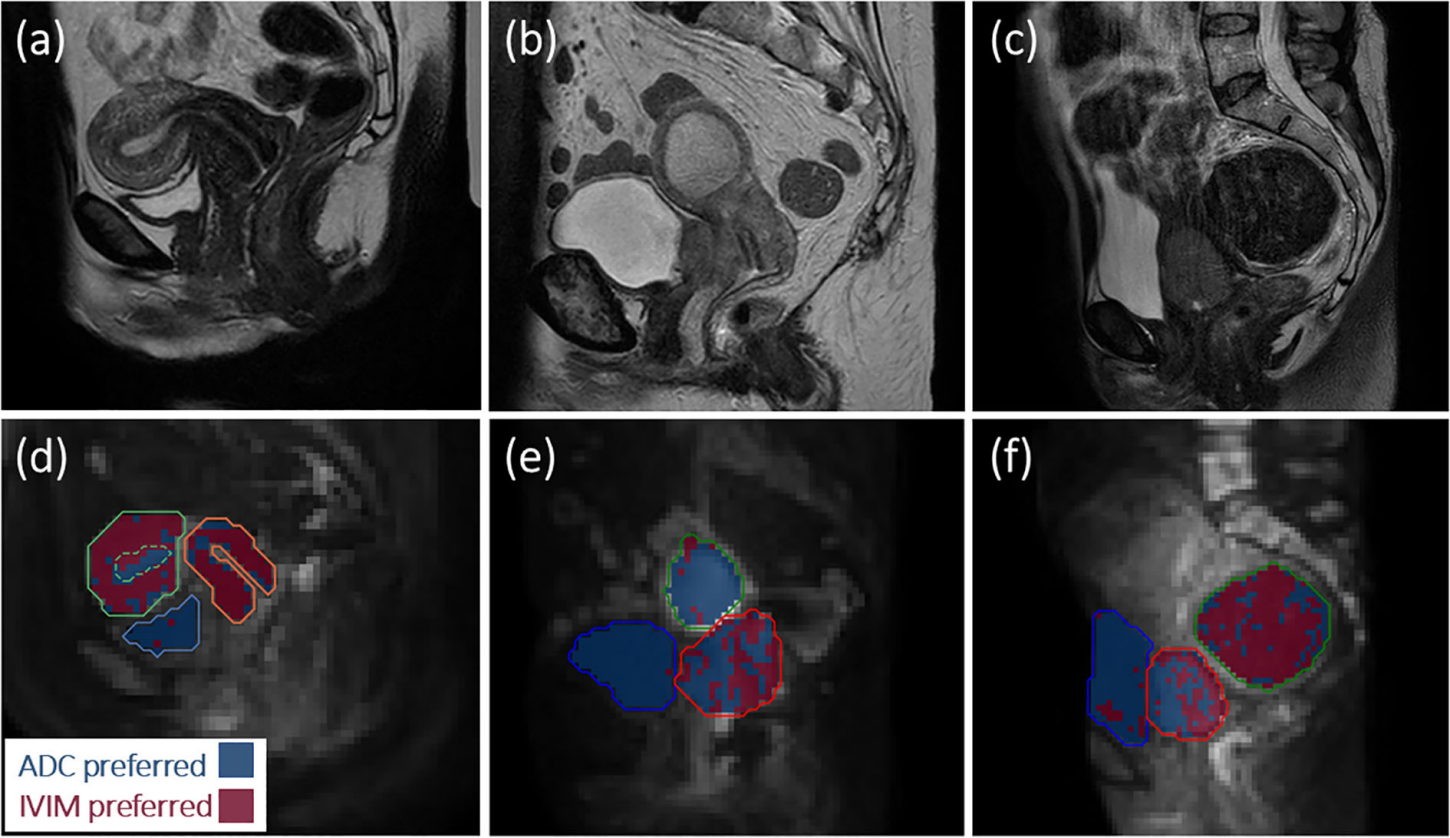
Anatomical images **(a-c)** and model preference maps overlaid on *b* = 800 s/mm^2^ images **(d-f)** for **(a, d)** one healthy volunteer, and **(b, e)**, **(c, f)** two patients. In **(d–f)**, blue represents voxels where the ADC model is preferred and red represents voxels where IVIM is preferred. For the healthy volunteer in **(d)**, ADC is preferred in the bladder (blue contour) and uterine fluid (green dashed contour), while IVIM tends to be preferred throughout the cervix (orange contour) and myometrium/junctional zone (green contour). For the patient in **(e)**, ADC is preferred in the bladder (blue contour) and uterine fluid (green contour), while there is spatial variation in the preferred model throughout the tumour (red contour). For the patient in **(f)**, ADC is preferred in the bladder (blue contour), IVIM tends to be preferred in the fibroid (green contour), and there is spatial variation in the preferred model throughout the tumour (red contour).

Example model preference maps (**Figure 1**) illustrate that ADC tends to be favoured in the bladder and uterine cavity fluid, while IVIM tends to be favoured in the uterine myometrium, cervix and in a fibroid. In tumours, there was spatial variation in the preferred model and large inter-patient variability in the proportion of voxels within individual tumours where IVIM better describes signal decays, with *p*_IVIM_ ranging from 0.13 to 0.68 across tumours at baseline.

### 3.2 Healthy volunteer repeatability and patient comparison

**Figure 2:**
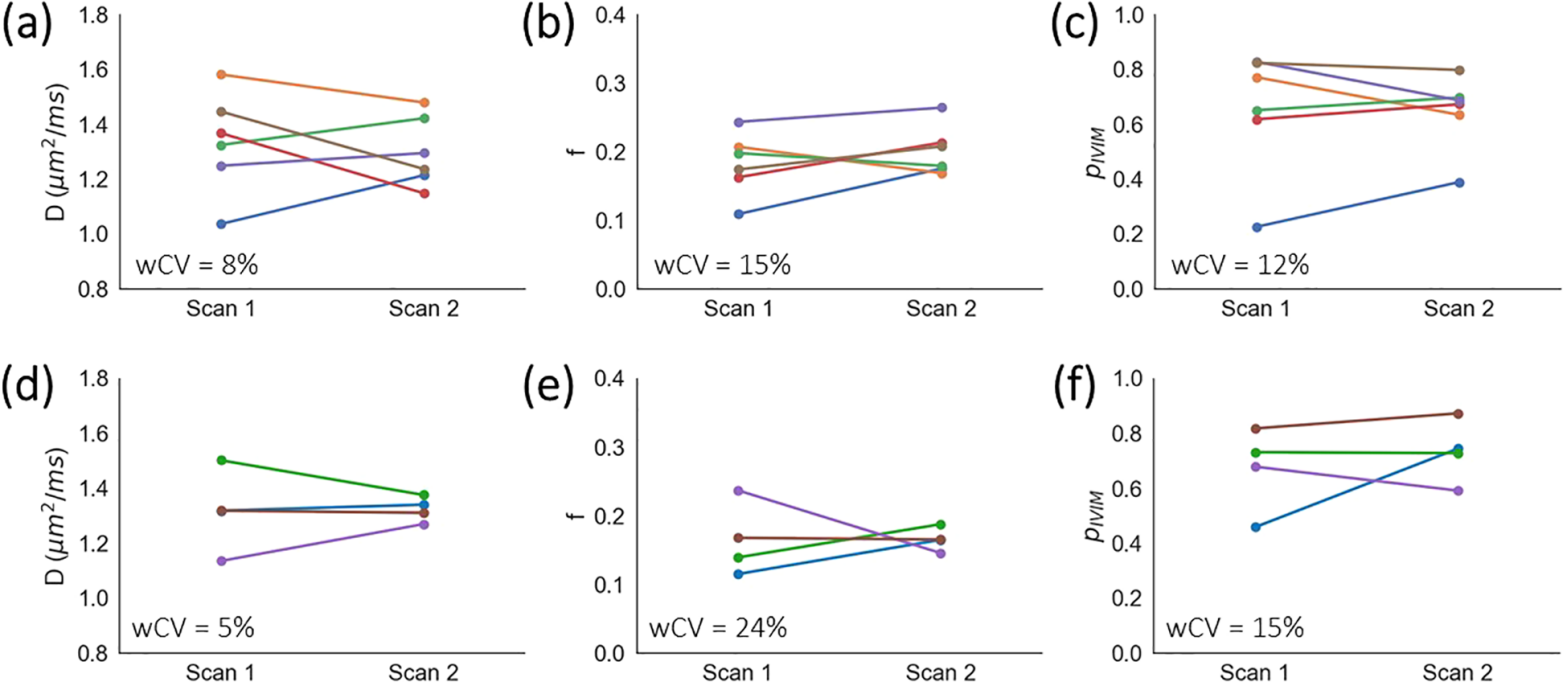
Healthy volunteer repeatability. Median *D*, median *f*, and *p*_IVIM_ values are plotted for repeat scans for **(a–c)** cervix and **(d–f)** uterine body ROIs. Each colour represents an individual healthy volunteer.

In the cervix (**Figures 2a-c**), mean ± s.d. across median values for all subjects and repeat scans were *D* = 1.3 ± 0.2 µm^2^/ms, *f* = 0.19 ± 0.04, and *p*_IVIM_ = 0.65 ± 0.18. The respective wCV values were 8%, 15%, and 12%. For the uterine body (**Figures 2d–f**), two volunteers were excluded from the repeatability analysis because in one of the two scans motion caused the uterine body to be misaligned across *b*-values. For the remaining four volunteers, *D* = 1.3 ± 0.1 µm^2^/ms, *f* = 0.17 ± 0.04, and *p*_IVIM_ = 0.70 ± 0.13. The respective wCV values were 5%, 24%, and 15%. For patients, one tumour dataset and three uterine body datasets were excluded due to image artefacts from signal ghosting (n = 2) and bulk patient motion (n = 2), following a qualitative review of image quality No evidence of violations of normality (Shapiro-Wilk p > 0.05) was found.

Significantly lower *D* (*p* = 0.00004) and lower *f* (*p* = 0.001) were observed in tumours compared with healthy cervix tissue (**Supplementary Figure S2a**). There was also a trend towards lower *p*_IVIM_ in tumours, but this was not significant (*p* = 0.160). In the uterine body (**Supplementary Figure S2b**), significantly lower *D* (*p* = 0.002) was observed in patients compared with healthy volunteers, while no significant differences were observed in *f* (*p* = 0.803) or *p*_IVIM_ (*p* = 0.183).

### 3.3 Sensitivity to treatment-induced biomarker changes in patient tumours

Three patients were excluded from the longitudinal analysis: one due to image artefact (motion resulting in significant misalignment across *b*-values), and two due to missing the week 3 scan.

Except for tumour volume at week 5 (Shapiro-Wilk *p* = 0.030), and ADC*_b_*_0_ at weeks 3 and 5 (Shapiro-Wilk *p* = 0.027 for both) no evidence of violations of normality or sphericity were found for any other parameter or time point. As IVIM parameters are the focus of this study, for simplicity it was decided to use the parametric repeated measures ANOVA for all data.

**Figure 3:**
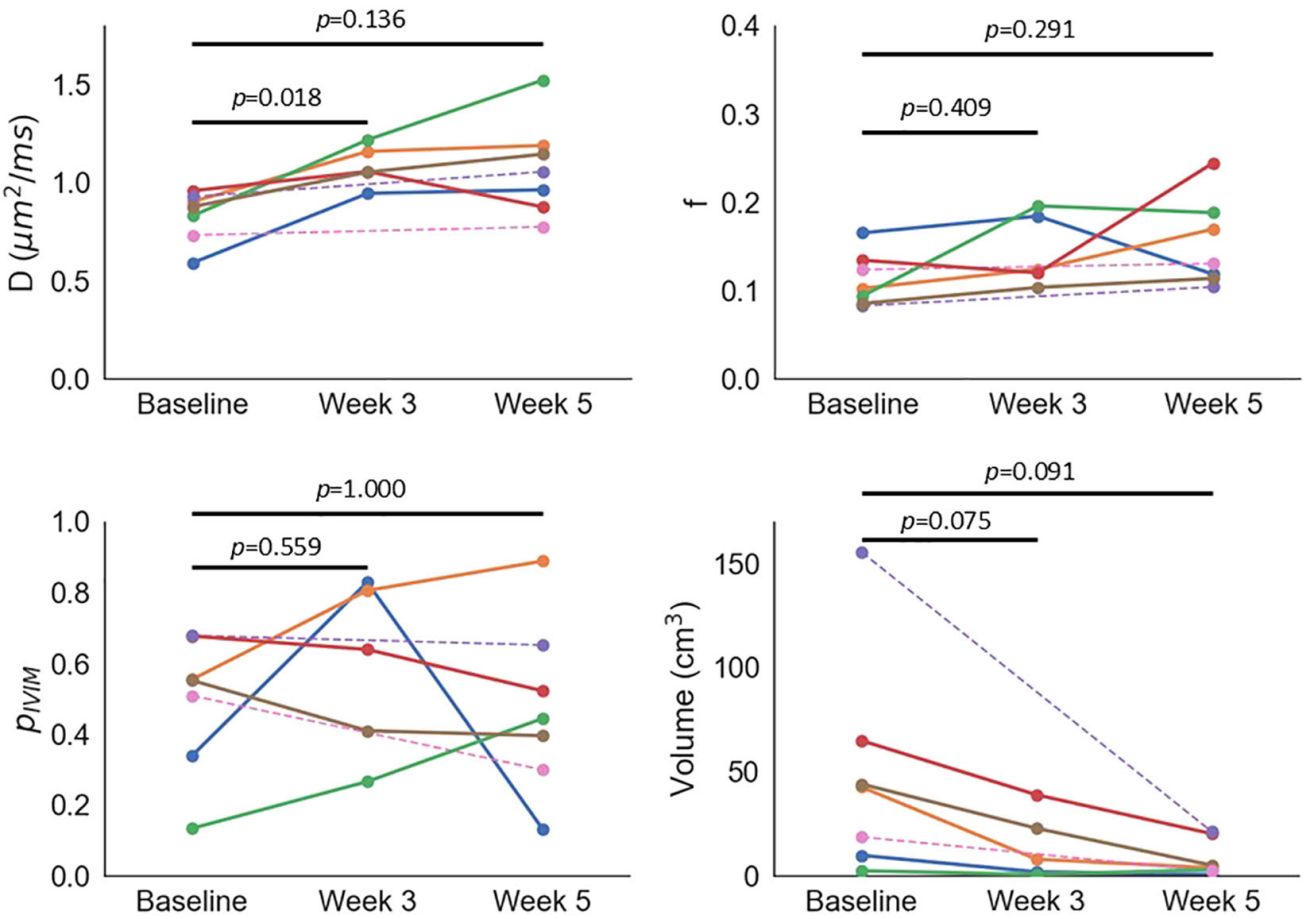
Median *D*, median *f*, *p*_IVIM_, and tumour volume as a function of time. Each colour represents an individual patient; dashed lines are used for two patients who did not have a scan at week 3.

ANOVA showed significant changes in *D* (*p* = 0.020) and tumour volume (*p* = 0.013), with non-significant changes in *f* (*p* = 0.226) and *p*_IVIM_ (*p* = 0.550) (**Figure 3**, **Supplementary Figure S3**). The only significant *post-hoc* test showed that *D* increased from baseline to week 3 (*p* = 0.018); *post-hoc* uncorrected *p*-values indicated significant tumour volume decreases from baseline to week 3 (*p* = 0.037) and week 5 (*p* = 0.045), but Bonferroni correction rendered these non-significant (*p* = 0.075 and 0.091, respectively). ANOVA showed a significant change in longitudinal tumour ADC*_b_*_150_ (*p* = 0.038) and ADC*_b_*_0_ (*p* = 0.010), but all Bonferroni-corrected *post-hoc* tests were not significant (all *p* > 0.08).

### 3.4 Influence of model preference on tumour IVIM and ADC parameters

When comparing median values from whole tumour ROIs with those from only the voxels where the IVIM model is favoured, *D* did not differ significantly at any time point (t-test *p* = 0.087, 0.490, 0.148, for baseline, week 3, and week 5, respectively). *f* was significantly higher in IVIM-favoured voxels at baseline and week 5 (t-test *p* = 0.039 and 0.012), but not at week 3 (Wilcoxon *p* = 0.063); with Bonferroni correction due to the three separate tests, only *f* at week 5 remains significant (*p* = 0.037). When comparing ADC_b0_ and *D*, ADC_b0_ was significantly higher at baseline and week 5 (t-test *p* = 0.004 and 0.004), but not different at week 3 (t-test *p* = 0.13); baseline and week 5 remain significant after Bonferroni correction due to the six separate tests (*p* = 0.027 and 0.025). When comparing ADC_b150_ and *D*, there was no significant difference at any time point (t-test *p* = 0.070, 0.177, 0.268).

### 3.5 Influence of perfusion on tumour diffusivities

**Figure 4:**
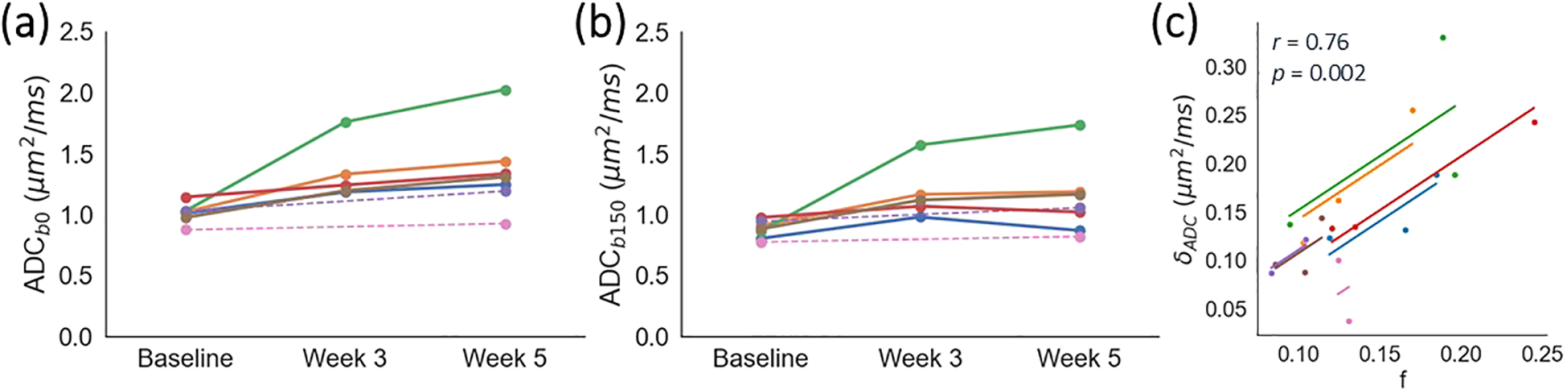
Impact of *b*-values on ADC. Median ADC calculated using minimum *b*-values of **(a)** 0 and **(b)** 150 s/mm^2^, as a function of time. Each colour represents an individual patient; dashed lines are used for two patients who did not have a scan at week 3. **(c)** The difference in ADC, δ_ADC_ = ADC*_b_*_0_ - ADC*_b_*_150_, correlates positively with *f* from IVIM. Each colour represents an individual patient, with data points showing median values of δ_ADC_ and *f*, and solid lines showing per-patient correlations. The cohort-level repeated measures correlation coefficient and associated *p*-value are shown in the plot.

ADC*_b_*_0_ was consistently higher than ADC*_b_*_150_, with δ_ADC_ = 0.15 ± 0.07 µm^2^/ms. δ_ADC_ correlated positively with *f* from IVIM (r = 0.76, *p* = 0.002; **Figure 4**). Absolute values of ADC*_b_*_0_ and ADC*_b_*_150_ correlated positively with *f* (r = 0.65, *p* = 0.015 and r = 0.62, *p* = 0.024, respectively; **Supplementary Figures S4a, b**). Conversely, *D* did not correlate with *f* (r = 0.35, *p* = 0.25; **Supplementary Figure S4c**).

## 4 Discussion

This work contributes to DW-MRI biomarker validation by showing that both IVIM- and ADC-favoured voxels exist within tumours; IVIM parameters are repeatable in healthy tissue and differ from those in tumours; *D* is sensitive to treatment-induced tumour changes; and that ADC values can suffer from a perfusion-dependent bias. The model comparison has shown that not all tumour tissue is best described by the IVIM model, *p*_IVIM_ has been introduced as a novel biomarker, and the bias in ADCs calculated with *b* = 0 s/mm^2^ has been shown to depend on *f*.

The model preference maps reflect expected trends based on the physiological characteristics of different tissues. In tumours, where intra- and inter-lesion microenvironment heterogeneity is expected, spatial variation and large inter-patient variability in model preference was observed. This shows that one MR signal model may not be applicable in all tumour voxels at all time points, highlighting that statistical model comparison should be considered when validating imaging biomarkers. Note that the accuracy of model comparison approaches has been shown to decrease with lower signal-to-noise ratios (SNR) (21), which may lead to an underestimation of *p*_IVIM_. This is an inherent limitation, with simpler models being increasingly preferred as data becomes noisier, which in this context would lead to ADC being preferred over IVIM. Future work could incorporate SNR into the model comparison, and incorporate the quantitative difference between models’ AIC_c_ values (24), to further assess the bias and precision of *p*_IVIM_. Nevertheless, the observed qualitative consistency between the model preference maps and known tissue characteristics provides evidence that the model comparison provides biologically meaningful information.

*D*, *f*, and *p*_IVIM_ exhibited good repeatability in the cervix of healthy volunteers, with *D* being the most repeatable. *D* was also the most repeatable in the uterine body, though for all parameters repeatability was poorer in the uterine body compared to the cervix. This may be partly due to the uterine body being more susceptible to inter-*b*-value misalignment, stemming from bladder filling and bowel motion. These effects should be more pronounced in the healthy volunteers, as Buscopan was not administered to help suppress bowel motion. Parameter estimates may also be affected by partial voluming with the endometrium and endocervical canal. For both ROIs, some variability may also be due to the repeated scans being performed at different times in the subjects’ menstrual cycle, which influences diffusion in uterine and cervical tissue (34, 35). Repeatability would therefore be expected to improve if repeat scans were performed in the same menstrual phase, with values reported here reflecting a worst-case scenario. Nevertheless, wCV for IVIM biomarkers were similar to estimates of ADC repeatability (36, 37) and lower than those derived from DCE-MRI (38, 39).

*D* and *f* differed significantly between healthy volunteers’ cervix tissue and patients’ cervical tumours, consistent with previous reports (9). Absolute values also agree well (9), though differences in sequence parameters, healthy volunteers’ age, and patients’ disease stage confound a direct comparison between studies. In the present study, the healthy volunteer cohort was younger than the patient group, and all healthy volunteers were pre-menopausal, which may contribute to the significant difference in uterine body *D* (40). As healthy cervix *D* has been reported to not vary with age (41), and significantly lower *D* has been reported in cervix tumours compared with age-matched healthy volunteers’ normal cervix (9), we expect age differences to be a minor factor in our comparison of healthy volunteers’ cervix and patients’ cervical tumours.*D* was the only parameter which showed sensitivity to treatment-induced changes, with an increase in *D* consistent with IVIM findings (13), and with ADC increasing following therapy (42). An increase in *D* may reflect a loss of cell membrane integrity and/or decreases in cell density as a result of chemoradiation (13), with some evidence that on-treatment *D* changes differ between responders and non-responders (14). Increases in *f* have been reported previously (13), but this was not observed in the present study. This may be due to the small patient numbers and greater variability of *f* compared with *D*, as indicated by the higher wCV in healthy volunteers. Changes in *f* are expected to reflect chemoradiation-induced changes in tumour microvasculature, with higher *f* values, suggesting higher perfusion, reported for responders than non-responders (14). Tumour *p*_IVIM_ also did not change significantly throughout treatment; as for *f*, this may be due to small patient numbers and poorer repeatability than *D*, based on healthy volunteer wCVs. *p*_IVIM_ is introduced here as a novel biomarker, with no previous reports in the literature, but we hypothesise that *p*_IVIM_ may have sensitivity to treatment if this induces changes in the proportions of distinct tumour microenvironments. While menopausal status has been shown to impact absolute values of DW-MRI tumour biomarkers (43), we are not aware of literature showing it has an impact on treatment-related changes, so do not consider this a confounding factor here.

As tumour volume tended to decrease throughout treatment, contouring was challenging at week 5; this is consistent with a previously reported increase in inter-observer variability in cervical tumour delineations post-treatment relative to baseline (17). Future studies may benefit from an earlier final time point. To complement the healthy volunteer repeatability reported in this work, future studies evaluating tumour biomarker repeatability are warranted, and could help determine biomarker sensitivity to treatment-induced changes (44). A ‘coffee-break’ assessment of short-term repeatability (45) may be beneficial here, removing the need for additional patient visits.

The AIC_c_ model comparison showed that the IVIM model is not favoured in all tumour voxels. This is expected to reflect a combination of noise, which can result in the simpler ADC model being favoured, and genuine spatial variation in the tumour microenvironment (46). ADC-favoured voxels are hypothesised to reflect regions without a measurable perfusion component, which could be due to the presence of necrosis or oedema, or areas of viable tumour with a very low vascular volume fraction. The model comparison shows that one signal model may not be applicable in all tumour voxels at all time points, highlighting that statistical model comparison should be considered when validating imaging biomarkers. Moreover, not accounting for model suitability can lead to a bias in summary model parameters, as shown here with a tendency for lower median *f* when including all tumour voxels, compared with only including voxels where IVIM is favoured. In addition, model suitability can impact diffusivities in voxels favoured by the ADC model, as shown here with a tendency for ADC_b0_ to yield higher diffusivities than *D*. The optimal approach to reporting summary statistics when there is spatial variability in model suitability requires further investigation; one option would be to only calculate parameter summary statistics from voxels where that parameter’s associated model has been selected.

For clinical translation, the fitting and model comparison pipeline would need to be incorporated into scanner and/or dedicated post-processing software. ADC maps are routinely generated on scanners, and this processing could be extended to IVIM parameter maps, acknowledging the added complexity of fitting a bi-exponential model (29). In terms of data acquisition, IVIM scan times will be longer than for ADC, though with further optimisation of *b*-values this time could be reduced (29). Overcoming these technical and practical challenges would aid the translation of IVIM-based biomarkers into clinical practice; for example as predictors of treatment response, as spatial maps to include in biological-image guided adaptive radiotherapy planning (47), or as early indicators of treatment response to guide subsequent treatment approaches. Such translation is supported by existing studies showing associations between pre-treatment IVIM biomarkers and RECIST-based treatment response (17, 48), correlations between IVIM biomarkers and a histology-derived hypoxia biomarker (49), and therapy-induced IVIM biomarker changes differing between responders and non-responders (13, 15).

Comparing ADC*_b_*_150_ and ADC*_b_*_0_ highlighted the impact of *b*-value selection on ADC, with perfusion effects increasing ADC when low *b*-values are included in the fit. This is especially relevant for cervical tumours, as several studies (3–7) include *b* = 0 s/mm^2^ and therefore introduce a bias in ADC. Importantly, the magnitude of this bias depends on the perfusion fraction, *f*, and if *f* changes throughout treatment, the ADC bias will change too. As such, it is recommended to not include *b* = 0 s/mm^2^ when evaluating cervical tumour ADC, to reduce the sensitivity to perfusion effects. Here, a pragmatic approach was taken to use a single *b*-value threshold of 150 s/mm^2^, but the degree to which this suppresses perfusion effects will depend on the underlying perfusion characteristics. This is a limitation of using a single threshold, and for tissue with significant and/or changing perfusion characteristics, it may be more appropriate to model this explicitly using IVIM. This is supported by our results showing that while ADC_b150_ has a weaker correlation with *f* than ADC*_b_*_0_, a correlation does still exist; moreover, use of *D* removes this correlation.

The main limitation of this study is the small sample size, for both healthy volunteers and patients. The small numbers reflect the exploratory nature of the trial from which these data come, and the results can be used to power follow-on studies. Further limitations are the lack of control for subjects’ menstrual cycles, and patient choice in having Buscopan to counteract bowel motion, which may be sources of variability in the reported biomarkers. Also, patients and healthy volunteers were not aged-matched, tumour repeatability was not assessed. The single-centre nature of the study limits the extent of technical validation that the study provides, and the study does not address direct biological validation as correlations between imaging and histology (e.g. cell and vessel densities) were not investigated. As the model comparison framework is based on IVIM and ADC model equations, we envisage it being readily applied to PGSE-based DW-MRI data from other vendors and/or field strengths. Future work can therefore extend the technical, biological, and clinical validation of IVIM biomarkers through multi-centre repeatability and reproducibility studies, imaging-histology correlations, and relating imaging biomarkers to outcomes.

Taken together, this study further advances the validation of DW-MRI biomarkers in cervical cancer. The model comparison demonstrated that the IVIM model is not favoured in all tumour voxels, indicating the presence of qualitatively different tumour microenvironments, and further demonstrating that model suitability should be evaluated as part of imaging biomarker validation. IVIM biomarkers were repeatable in healthy tissue, with *D* sensitive to early therapy-induced changes in tumours. In addition, ADC calculated from low *b*-values suffers from a perfusion-dependent bias.

## Data Availability

The raw data supporting the conclusions of this article will be made available by the authors, without undue reservation.
