# Supplementary material for: Intra-voxel incoherent motion biomarker repeatability in healthy volunteers and sensitivity to chemoradiotherapy-induced changes in patients with uterine cervical cancer
Source: Front Oncol. 2025 Nov 26;15:1633456. doi: 10.3389/fonc.2025.1633456 (PMC12689363; doi:10.3389/fonc.2025.1633456)
Supplement: Supplementary file 1 [file DataSheet1.pdf]

Supplementary Material

1 Supplementary Table

Supplementary Table 1. Patient characteristics

ID	Age (years)	PS	Stage	Histology	Grade	LVSI	Pelvic nodes
**1**	51	0	IIB	squamous	2	absent	no
**2**	73	0	IVA	squamous	3	absent	yes
**3**	77	1	IB2	adenocarcinoma	1	not known	no
**4**	30	0	IIIC1	squamous	3	present	yes
**5**	37	0	IIIC1	squamous	2	not known	yes
**6**	46	0	IIB	squamous	3	absent	no
**7**	36	0	IIB	squamous	2	absent	no
**8**	28	0	IVA	squamous	2	present	no

PS, Performance status; LVSI, lymphovascular space invasion.

2 Supplementary Figures

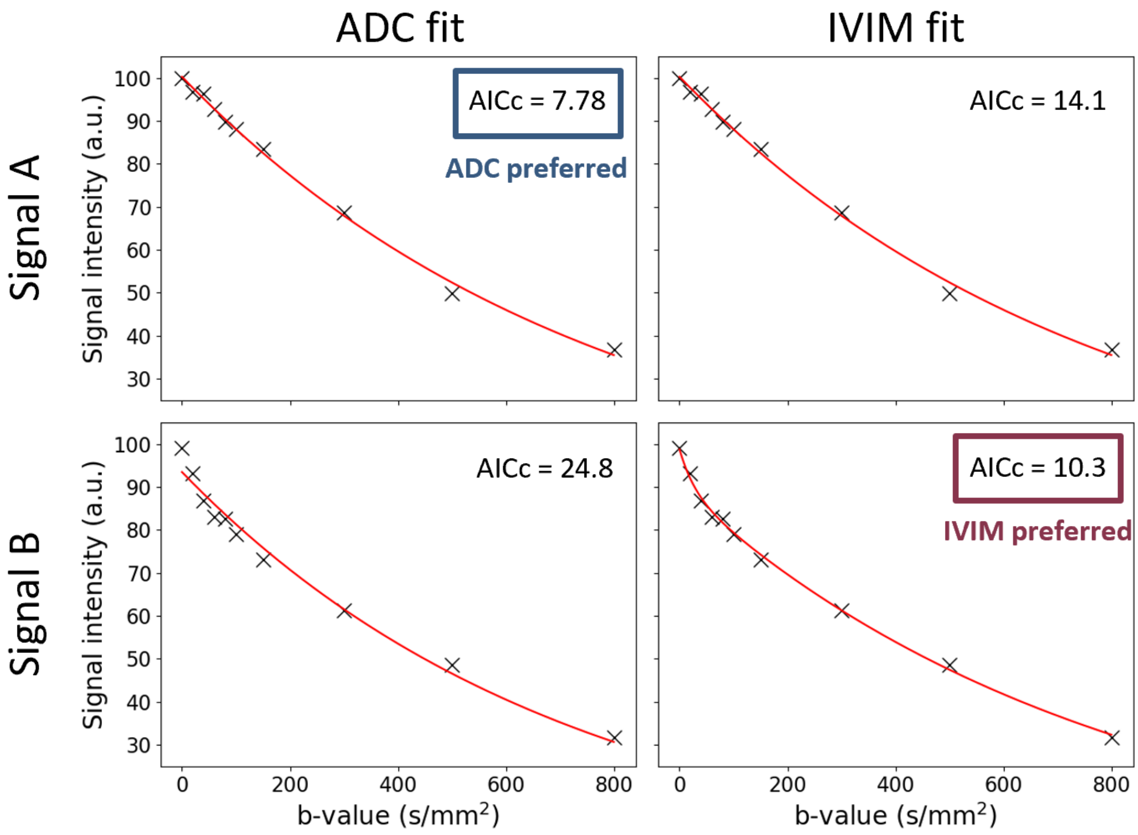

Supplementary Figure 1. Model comparison schematic. ADC and IVIM models are fitted to two simulated signals, A and B. For a given signal, AIC_c values are calculated for each fit and the fit giving the lowest AIC_c is selected as the preferred model. Here, ADC is preferred for signal A, and IVIM is preferred for signal B.

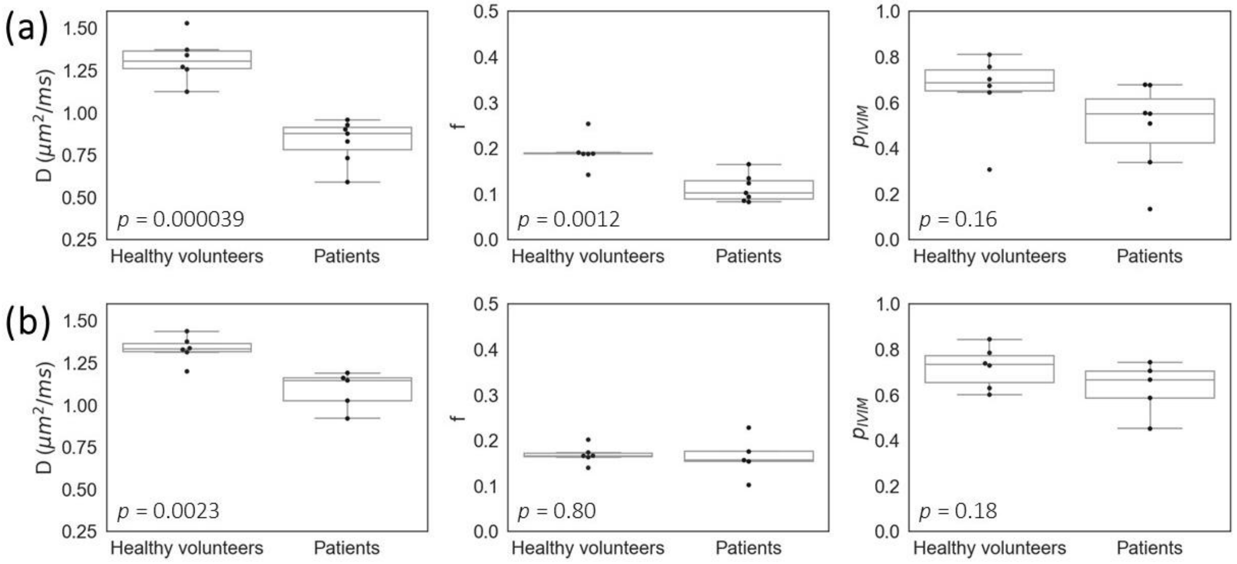

Supplementary Figure 2. Comparison between healthy volunteers and patients. Parameters are shown for (a) healthy volunteer cervix and patient tumours and (b) healthy volunteer and patient uterine body ROIs. The unpaired t-test p-value is shown for each parameter.

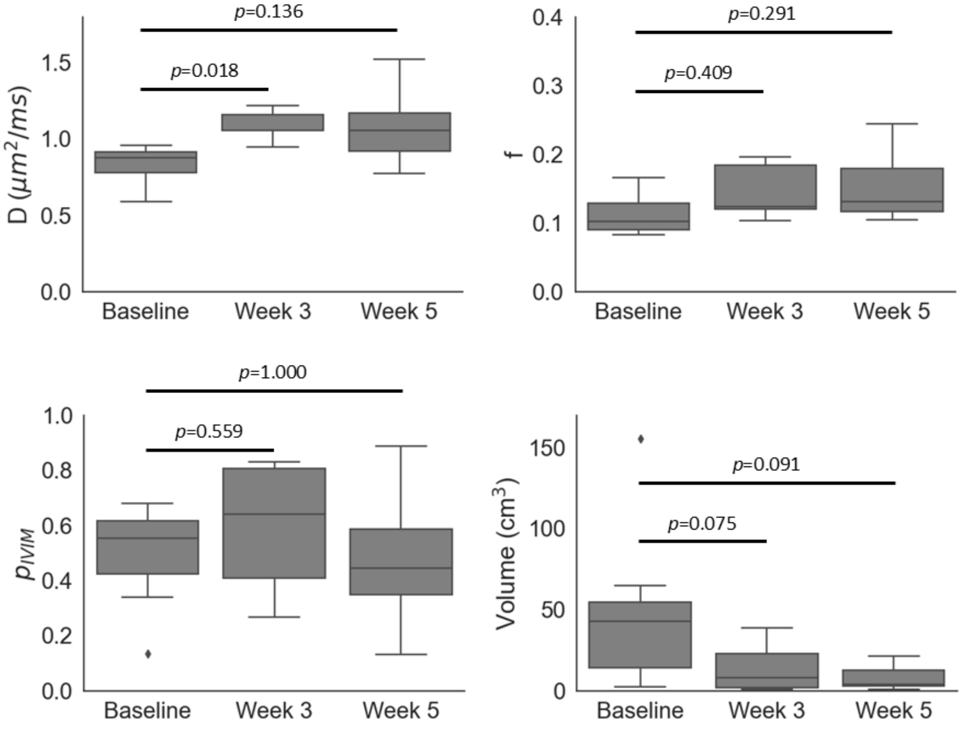

Supplementary Figure 3. Boxplots for median D, median f, p_{IVIM} and tumour volume as a function of time. This plots the same data as shown in Figure 4 in the main text. Bonferroni-corrected p-values are shown for post-hoc tests following repeated measures ANOVA.

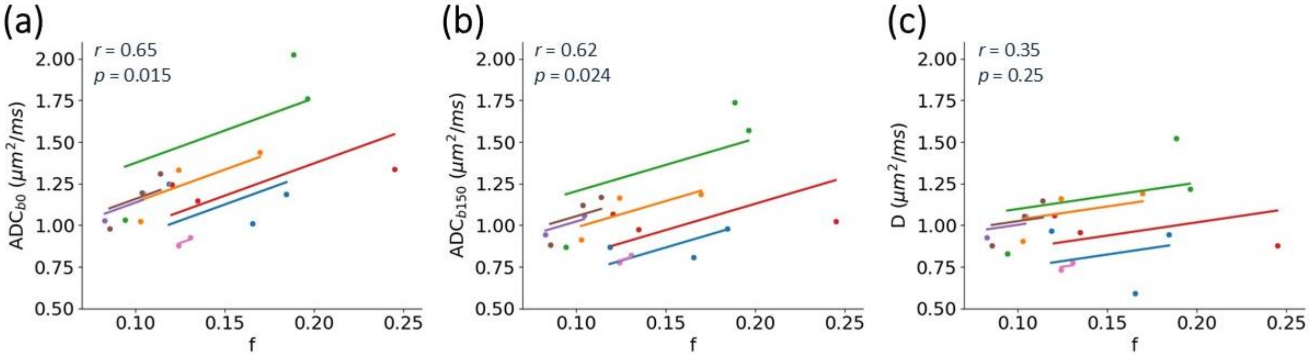

Supplementary Figure 4. Repeated measures correlations between f and (a) ADC$_{b0}$, (b) ADC$_{b150}$, and (c) D. Each colour represents an individual patient, with data points showing median values of diffusivities and f, and solid lines showing per-patient correlations. The cohort-level repeated measures correlation coefficient and associated p-value are shown in each plot.
